# The Bactericidal Activity of Carbon Monoxide–Releasing Molecules against *Helicobacter pylori*

**DOI:** 10.1371/journal.pone.0083157

**Published:** 2013-12-26

**Authors:** Ana F. Tavares, Margarida R. Parente, Marta C. Justino, Mónica Oleastro, Lígia S. Nobre, Lígia M. Saraiva

**Affiliations:** 1 Department of Biological Chemistry, Instituto de Tecnologia Química e Biológica, Universidade Nova de Lisboa, Oeiras, Portugal; 2 Department of Infectious Diseases, Instituto Nacional de Saúde Dr Ricardo Jorge, Lisboa, Portugal; Institut Pasteur Paris, France

## Abstract

*Helicobacter pylori* is a pathogen that establishes long life infections responsible for chronic gastric ulcer diseases and a proved risk factor for gastric carcinoma. The therapeutic properties of carbon-monoxide releasing molecules (CORMs) led us to investigate their effect on *H. pylori*. We show that *H. pylori* 26695 is susceptible to two widely used CORMs, namely CORM-2 and CORM-3. Also, several *H. pylori* clinical isolates were killed by CORM-2, including those resistant to metronidazole. Moreover, sub-lethal doses of CORM-2 combined with metronidazole, amoxicillin and clarithromycin was found to potentiate the effect of the antibiotics. We further demonstrate that the mechanisms underpinning the antimicrobial effect of CORMs involve the inhibition *of H. pylori* respiration and urease activity. *In vivo* studies done in key cells of the innate immune system, such as macrophages, showed that CORM-2, either alone or when combined with metronidazole, strongly reduces the ability of *H. pylori* to infect animal cells. Hence, CORMs have the potential to kill antibiotic resistant strains of *H. pylori*.

## Introduction

*Helicobacter pylori* is a pathogen that colonizes the gastric mucosa of humans and is ubiquitous in over half the world’s population. Once acquired, *H. pylori* establishes lifelong infections that are the major cause of gastric and duodenal ulcer diseases and malignant gastric cancer [1]. *H. pylori* uses several factors that enable colonization [2]. In particular, the activity of the nickel-dependent urease is essential for the survival and pathogenesis of the bacterium as this enzyme hydrolyses urea to ammonia, which neutralizes the stomach acidity [3], [4]. The more widely used antibiotics for treatment of *H. pylori* are metronidazole, clarithromycin, amoxicillin and tetracycline [5]. However, when given as monotherapy none of these drugs are able to eradicate *H. pylori*. Hence, infections with *H. pylori* are usually treated with a combination of drugs, which consists of two or three antibiotics together with an acid-suppressive drug (a proton pump inhibitor, e.g. omeprazole) [5]. Yet, the efficacy of these multiple antibiotic therapies is decreasing mainly due to the crescendo occurrence of antibiotic-resistant *H. pylori* strains. In particular, metronidazole resistant strains are a major cause of *H. pylori* treatment failure [6].

The endogenous production of carbon monoxide (CO), via the mammalian heme oxygenase (HO), exerts benefits in the neural, cardiovascular and renal systems [7]. Moreover, it has remarkable impact on microbial sepsis as HO-1 deficient mice display enhanced susceptibility to polymicrobial infections, and the administration of exogenous CO rescues the HO-1-deficient mice from sepsis-induced lethality [8], [9]. The CO-releasing molecules (CORMs) are metal carbonyls capable of transferring CO directly to a target within a cell, which have been shown to represent a more effective therapeutic way to deliver CO in mammals and with potential for several medical applications [10]–[12]. The release of CO from CORMs is triggered by light exposure, pH variation or through ligand substitution. In particular, the ruthenium-based carbonyl CORM-2 promotes carboxyl-myoglobin formation upon dissolution in dimethyl sulfoxide, with a half time of ∼1 min. CORMs have been also reported to be stable molecules at low pH, which represents an advantage for their utilization in acidic environments [13], [14]. In 2007, CORMs were reported to have antimicrobial properties against *Escherichia coli* and *Staphylococcus aureus* [15]. Since then, other bacteria such as *Pseudomonas aeruginosa* and *Mycobacterium tuberculosis* showed to be susceptible to CORMs [16], [17]. Importantly, Ru^2+^ complexes structurally similar to CORM-2 and CORM-3 but devoid of CO do not kill bacteria, an observation that is consistent with the inhibition of bacterial components by CO [15], [18]. However, CO alone is not as effective either, as the presence of the transition metal carbonyl is required to elicit the antimicrobial action, which relies on effects mediated by a network that involves CO liberation and ROS formation [19]. Due to the emergence of *H. pylori* resistance strains in this work we have tested the effectiveness of CORMs against *H. pylori* under *in* *vitro* conditions and during infection of mammalian cells.

## Materials and Methods

### Reagents

Tricarbonylchloro(glycinato)ruthenium(II) (CORM-3, Alfama) and tricarbonyldichlororuthenium(II) dimer (CORM-2, Sigma), used as CO donors, were freshly prepared by dissolution in water and dimethyl sulfoxide (DMSO), respectively. Dichlorotetrakis(dimethylsulfoxide)ruthenium(II) dissolved in DMSO (Strem chemicals) was used as the CO-depleted form of Ru-based CORM-2 (herein named iCORM-2), and used in a concentration twice the molar concentration of CORM-2. Metronidazole, amoxicillin, and clarithromycin (Sigma) were dissolved in water.

### Bacterial Strains, Growth Conditions and Viability Assays

*Helicobacter pylori* 26695 reference strain and six clinical strains, isolated from human gastric biopsies and belonging to the collection of Instituto Nacional de Saúde Doutor Ricardo Jorge, Portugal, were analysed. *H. pylori* strains were cultured, at 37°C, under a microaerobic atmosphere (6% O_2_, 7% CO_2_, 3.5% H_2_ and 83.5% N_2_) generated by an Anoxomat system (MART Microbiology). Growth was performed in 10% horse blood-agar (HBA, Oxoid) plates and in brain heart infusion (BHI, Oxoid) liquid medium, both supplemented with a cocktail of antibiotics/fungicide (12.5 mg/L vancomycin, 0.3 mg/L polymyxin B, 6.3 mg/L trimethoprim and 5.0 mg/L amphotericin B). The liquid medium was further supplemented with 10% (v/v) decomplemented fetal calf serum (FCS, Gibco-Invitrogen) or with 0.2% β-cyclodextrin (βCD, Sigma).

To determine the susceptibility of *H. pylori* to CORMs, bacterial suspensions (prepared as in Protocol S1) were inoculated at an optical density at 600 nm (OD_600_) ∼0.05 in BHI-βCD liquid media and treated with CORM-3, CORM-2, iCORM-2 and/or metronidazole. The number of viable cells was evaluated by measuring the colony-forming unit per millilitre (CFU/mL) formed on HBA plates. When indicated, *H. pylori* growth was supplemented with 5 mM reduced glutathione (Sigma) or 5 mM cysteine (Fluka).

The minimal inhibitory concentration (MIC) and the minimal bactericidal concentration (MBC) were determined [20] for CORM-3, CORM-2, metronidazole, amoxicillin and clarithromycin (as described in Protocol S2).

### Oxygen Consumption Assays and Spectrophotometric Analysis of Cytochromes

Oxygen consumption assays were done in bacterial suspensions (prepared as detailed in Protocol S3) treated for 5 min with CORM-2 (25 mg/L), iCORM-2 (50 mg/L) or left untreated and stimulated by addition of sodium pyruvate (5 mM), and monitored in a Rank Broths oxygen electrode (Hansatech).

*H. pylori* cells grown for 24 h on HBA plates were resuspended in 20 mM Tris-HCl pH 7.5 and incubated with lysozyme for 30 min at 37°C. Cells were then centrifuged for 30 min at 12000 g to collect cell debris. The supernatants containing membranes and cytoplasm were reduced by addition of a saturated buffered solution of sodium dithionite and treated with 200 mg/L CORM-2, for 2 min. Spectra were recorded in a Shimadzu UV-1700 spectrophotometer and the reduced-plus-CORM-2 minus reduced difference spectra was calculated.

### Urease Activity Assays

Urease activity was determined in cellular suspensions of *H. pylori* grown, for 15 h, in the absence and in the presence of 200 mg/L CORM-2 or 400 mg/L iCORM-2. Also, cellular suspensions of *H. pylori* grown for 24 h on HBA plates in the absence of any carbon monoxide source were collected and exposed, for 15 min, to several concentrations of CORM-2. Urease activity was determined spectrophotometrically at 560 nm [21] in 50 µL cellular suspensions (prepared as described above and detailed in Protocol S3) that were incubated, for 30 min, with 500 µL 0.7 mM KH_2_PO_4_ Na_2_HPO_4_ (pH 6.9) buffer, 300 mM urea (Sigma) and 0.1 mM phenol red (Sigma).

### *Helicobacter pylori* Viability in Macrophages

Murine macrophage cell line RAW264.7 (ATCC Tib71) was seeded with 5×10^5^ cells per well, in 24-well plates (Sarstedt) containing Dulbecco’s modified Eagle’s medium (see Protocol S4) and grown for 3 h at 37°C in humidified 5% CO_2_ atmosphere. At this point, macrophages were activated with 0.3 µg/mL gamma interferon (IFN-γ, Sigma) for 12 h. Bacterial suspensions were used to infect macrophages cultured in infection medium, at a multiplicity of infection (MOI) of ∼100. After incubation for 3 and 6 h, at 37°C and 5% CO_2_, each well was scraping to evaluate the viable bacterial cells (see details in Protocol S4).

### Statistical Analyses

Statistical analyses were performed with GraphPad Prism 5 (GraphPad Software) using, as indicated, either One-way or Two-way ANOVA followed by a Bonferroni multiple comparison test. Analysis of the MIC and MBC data was done with the Mann Whitney *t* test considering the significance threshold at P<0.05 (95% confidence level). Data are presented as mean ± standard error (SE), with exception for MICs and MBCs represent medians.

## Results

### *Helicobacter pylori* Viability Is Inhibited by CORMs

**Figure 1:**
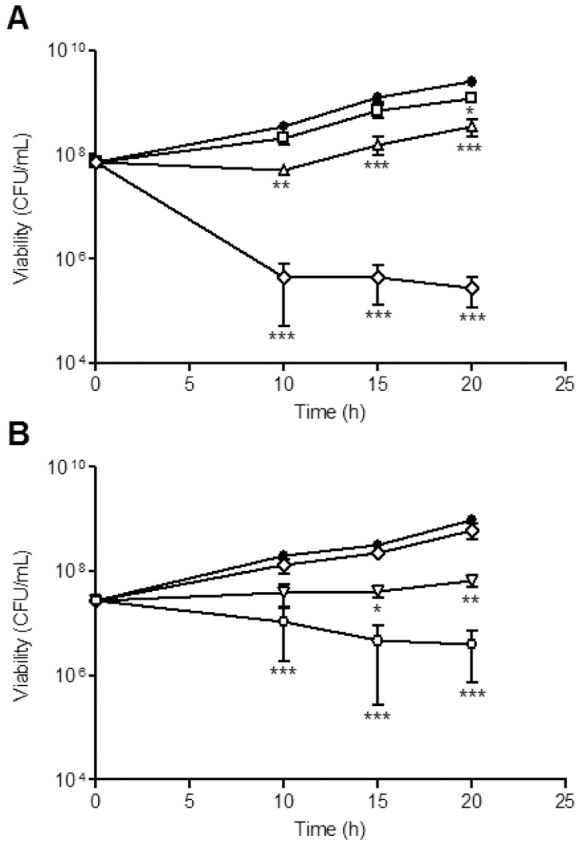
CORMs affect *H. pylori* viability. Cell viability of *H. pylori* 26695 left untreated (filled circle) and treated with 100, 150 and 200 mg/L CORM-2 (open square, triangle and diamond, respectively) (**A**), and exposed to 120, 240 and 300 mg/L CORM-3 (open diamond, inverted triangle and circle, respectively) (**B**). The number of viable cells were determined for four biological samples and are expressed as means ± SE. *p<0.05, **p<0.01 and ***p<0.001 (Two-way ANOVA and Bonferroni test).

To examine how CORMs affect the growth of *H. pylori* 26695, CORM-2 and CORM-3 were added to cultures growing in BHI-βCD under microaerobic conditions. Both CORMs inhibited viability during 20 h, in a concentration-dependent manner (Fig. 1). The effect mediated by CORM-2 was stronger than that exerted by CORM-3. Exposure of *H. pylori* to 200 mg/L CORM-2, for 15 h, induced a 4-log loss of cell viability (Fig. 1A), while treatment with 300 mg/L CORM-3 lowered the viability by 2-log (Fig. 1B). Interestingly, the decrease of the *H. pylori* counts caused by 300 mg/L CORM-2 was comparable to that induced by 1.5 mg/L metronidazole (Fig. 1A and S1). Values of 200 and 250 mg/L (CORM-2) and 420 and 510 mg/L (CORM-3) were obtained for the MIC and MBC, respectively. Furthermore, the ratio MBC/MIC was lower than 4 revealing the bactericidal character of the two drugs [22].

Some antibiotics were reported to stimulate the formation of coccoid forms [23], therefore, we tested whether CORMs promote similar modifications. However, even after 20 h exposure to 200 mg/L CORM-2, the shape of the treated cells remained unaltered and the presence of coccoid forms was not observed (data not shown).

As CORMs are ruthenium-containing compounds we have analysed whether the transition metal contributed to the antimicrobial properties by growing *H. pylori* in the presence of the CO-depleted compound but that still contains ruthenium, namely iCORM-2. However, up to 400 mg/L iCORM-2 elicited no growth impairment (Fig. S2), revealing that the metal does not interfere and CO is required for the bactericidal effect.

Since *H. pylori* is more effectively killed by CORM-2, the following experiments were performed with this compound.

### CORM-2 Impairs *H. pylori* Respiration

**Figure 2:**
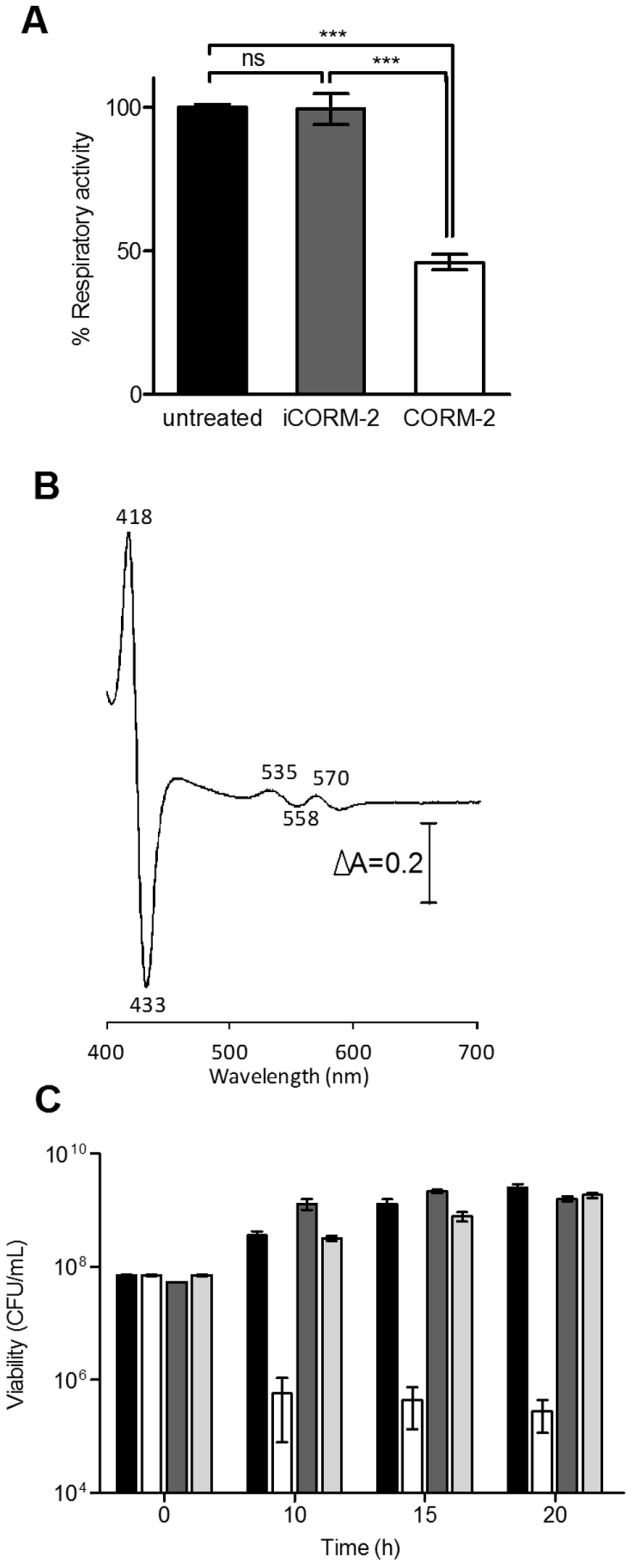
CORM-2 inhibits *H. pylori* respiration. (**A**) Cellular suspensions of *H. pylori* were left untreated (black) and treated for 5 min with 50 mg/L iCORM-2 (grey) and 25 mg/L CORM-2 (white). Oxygen consumption was measured, after the addition of pyruvate (5 mM) in a Clark-type O_2_ electrode for two biological samples of *H. pylori* assayed in triplicate. Values are expressed as means ± SE. ***p<0.001, ns - non significant (One-way ANOVA and Bonferroni test). (**B**) Spectral characterization of *H. pylori* cells treated with CORM-2. CORM-2 (200 mg/L) was added to reduce cells of *H. pylori* and the spectra recorded 5 min after addition of compound. Numbers indicate wavelength features (nm) in the difference reduced-plus-CORM-2 minus reduced spectrum. Two independent biological samples were analyzed. (**C**) Viability of *H. pylori* grown in BHI-βCD medium (black) and exposed to 200 mg/L CORM-2 alone (white), and in the presence of 5 mM glutathione (dark grey) or 5 mM cysteine (light grey). Values represent average of three biological samples with the respective SE.

Since CORM-2 showed to be an inhibitor of *H. pylori* growth, we sought whether CORM-2 inhibited cellular respiration. While *H. pylori* cells left untreated and stimulated with pyruvate had a considerable oxygen reductase specific activity (∼1.2 nmol O_2_/min/CFU), incubation with CORM-2 for 5 min caused a decrease of more than 50% in oxygen (Fig. 2A). Furthermore, the difference spectrum of *H. pylori* cells treated with CORM-2 (reduced-plus-CORM-2 minus reduced, Fig. 2B) shows a Soret band at 418 nm with a trough at 433 nm and bands at 535 and 570 nm, with a trough at 585 nm. These features are characteristic of the *in* *vivo* formation of carbonmonoxy adducts binding cytochrome *b* and *c*, and have been proposed to arise from the ligation of CO to *cbb3*-type cytochrome oxidase, so far the sole terminal oxidase cytochrome of *H. pylori* [24], [25].

We observed that addition of glutathione and cysteine prevents bacterial killing by CORM-2 (Fig. 2C). However, no formation of reactive oxygen species ROS could be detected and supplementation with ascorbic acid did not inhibit the CORM-2 antimicrobial action (data not shown). Although similar results were previously reported for CORM-3-treated *Pseudomonas aeruginosa*, the rational behind abolishment of the CORM bactericidal effect by thiol donors remains essentially unclear [16].

### CORM-2 Inhibits *H. pylori* Urease Activity

**Figure 3:**
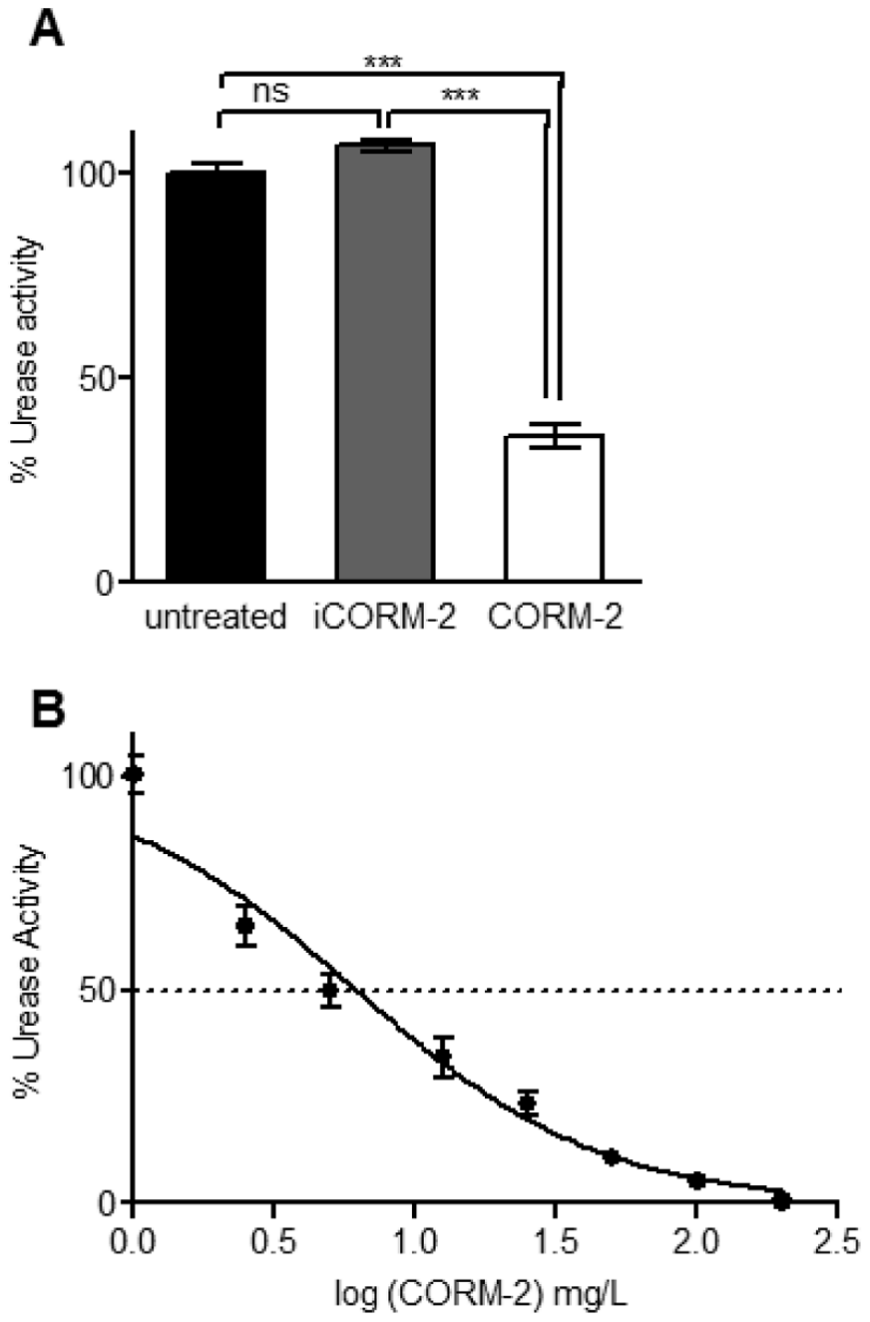
CORM-2 inhibits urease activity of *H. pylori.* (**A**) Urease activity was measured in *H. pylori* cells left untreated (black), treated for 15 h with 400 mg/L iCORM-2 (grey) and 200 mg/L CORM-2 (white). The results represent the average of three biological samples performed in duplicate, and error bars represent SE. ***p<0.001, ns - non significant (One-way ANOVA and Bonferroni test). (**B**) Urease activity of *H. pylori* cell suspensions treated, for 15 min, with CORM-2 (0, 2.5, 5, 12.5, 25, 50, 100 and 200 mg/L). The results are the average of five biological samples and error bars represent SE.

*H. pylori* expresses significant amounts of a nickel-containing urease, which is a key enzyme for its virulence [3]. Since CO is able to bind transition metals, we analysed the effect of CORM-2 in the urease activity in: i) cells grown in the presence of CORM-2 and ii) cells growth in the absence of the CO donor, collected and then exposed to several concentrations of CORM-2. The results showed that *H. pylori* grown in the presence of 200 mg/L CORM-2 exhibited a urease activity decrease of ∼65% (Fig. 3A). Also, the incubation of *H. pylori* cells with increasing concentrations of CORM-2, for 15 min, cause impairment of the urease activity. Using CORM-2 concentrations up to 200 mg/L, a value of half-maximal inhibitory concentration IC_50_ of 6±1 mg/L was determined (Fig. 3B).

### CORM-2 Is Able to Kill Metronidazole-Resistance *H. pylori* Strains

We investigated the toxicity of CORMs towards six *H. pylori* clinical strains with different degree of metronidazole resistance (Table 1). All these strains were isolated from patients suffering from non-ulcer dyspepsia, and present different antibiotic resistance profiles to metronidazole and clarithromycin. Strains 4574, 5587, 5611 and 5846 are resistant to both antibiotics, the 5599 strain is susceptible to metronidazole and resistant to clarithromycin and the 4597 strain is resistant to metronidazole and susceptible to clarithromycin. Additionally, they are all susceptible to amoxicillin.

**Table 1:** MICs of CORM-2 and metronidazole to the *H. pylori* reference strain 26695 and the indicated clinical isolates.

	MIC (mg/L)	
Strain	CORM-2	Metronidazole*
**26695**	200	8
**5599**	200	2
**5611**	150	64
**5846**	100	16
**4597**	200	32
**4574**	150	32
**5587**	100	32

Resistant (MIC>8 mg/L).

The clinical isolates showed different susceptibility to CORM-2, with MIC_CORM-2_ values ranging from 100 and 200 mg/L (Table 1). Moreover, CORM-2 acted as a bactericidal since the MBC/MIC ratios determined were lower than four (Table S1). While the highest MICs of CORM-2 (MIC_CORM-2_ = 200 mg/L) were observed for the metronidazole-susceptible strains, (Table S1), the growth of metronidazole-resistant clinical isolates (MIC_metronidazole_ >8 mg/L) was inhibited by lower concentrations of CORM-2 (MIC_CORM-2_≤150 mg/L). The only exception occurred for the metronidazole-resistant strain 4597, which exhibited the same MIC for CORM-2 than the metronidazole-susceptible strains (Table S1).

### Combination of CORM-2 and Antibiotics Attenuates *In Vitro H. pylori* Viability

**Figure 4:**
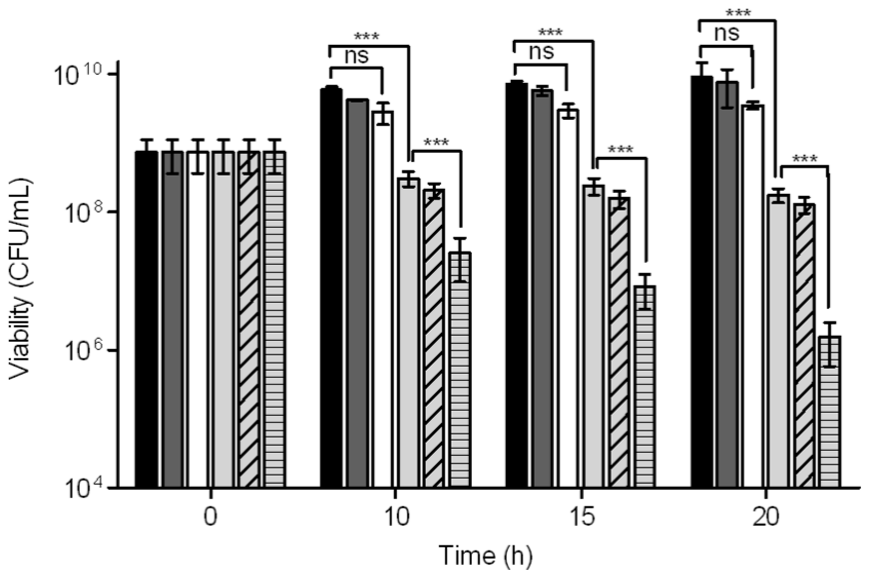
Effect of combined metronidazole and CORM-2 treatment on *H. pylori* viability. Cell viability of *H. pylori* 26695 left untreated (black) and exposed to iCORM-2 (dark grey), 100 mg/L CORM-2 (white), 1.5 mg/L metronidazole (light grey), 1.5 mg/L metronidazole plus 200 mg/L iCORM-2 (light grey, diagonal strips) and 1.5 mg/L metronidazole plus 100 mg/L CORM-2 (light grey, horizontal strips). The number of viable cells were determined for four independent cultures and are expressed as means ± SE. ***p<0.001 (Two-way ANOVA and Bonferroni test).

**Figure 5:**
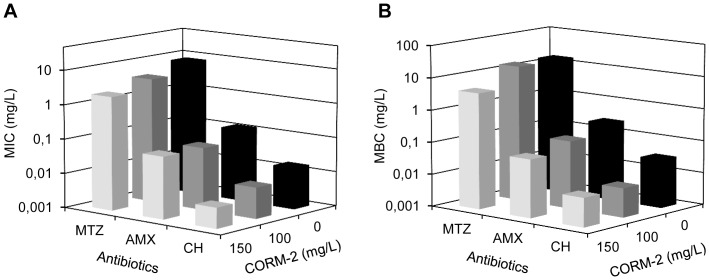
Effect of CORM-2 on MIC/MBC values of metronidazole, amoxicillin and clarithromycin for *H. pylori*. MICs (**A**) and MBCs (**B**) of metronidazole (MTZ), amoxicillin (AMX) and clarithromycin (CH) against *H. pylori* 26995 determined in the absence (black) and in the presence of 100 mg/L (dark grey) and 150 mg/L CORM-2 (light grey). Results represent the median of five biological samples and are significantly different in all cases (p<0.05 in Mann Whitney *t* test).

Since *H. pylori* infections are usually eradicated by means of triple or even quadruple therapies, we analysed the effect of CORM-2 as an adjuvant of the currently used antibiotics. For this purpose, *H. pylori* was treated with metronidazole, amoxicillin or clarithromycin combined with a sub-lethal dose of CORM-2. Non-significant loss of viability was observed upon exposure of *H. pylori* to metronidazole (1.5 mg/L) (≤2-log) or to CORM-2 (100 mg/L). However, simultaneous exposure to the two drugs resulted in an accentuated drop in recovered viable bacteria (∼4-log) (Fig. 4). The combination of CORM-2 and metronidazole translated into a reduction of 50% of the MIC for metronidazole (Fig. 5 and Table S2). Likewise, the combination of CORM-2 with amoxicillin and clarithromycin led to a significant decrease of both MIC and MBC that, similarly to metronidazole, was dependent on the CORM-2 concentration (Fig. 5 and Table S2).

**Figure 6:**
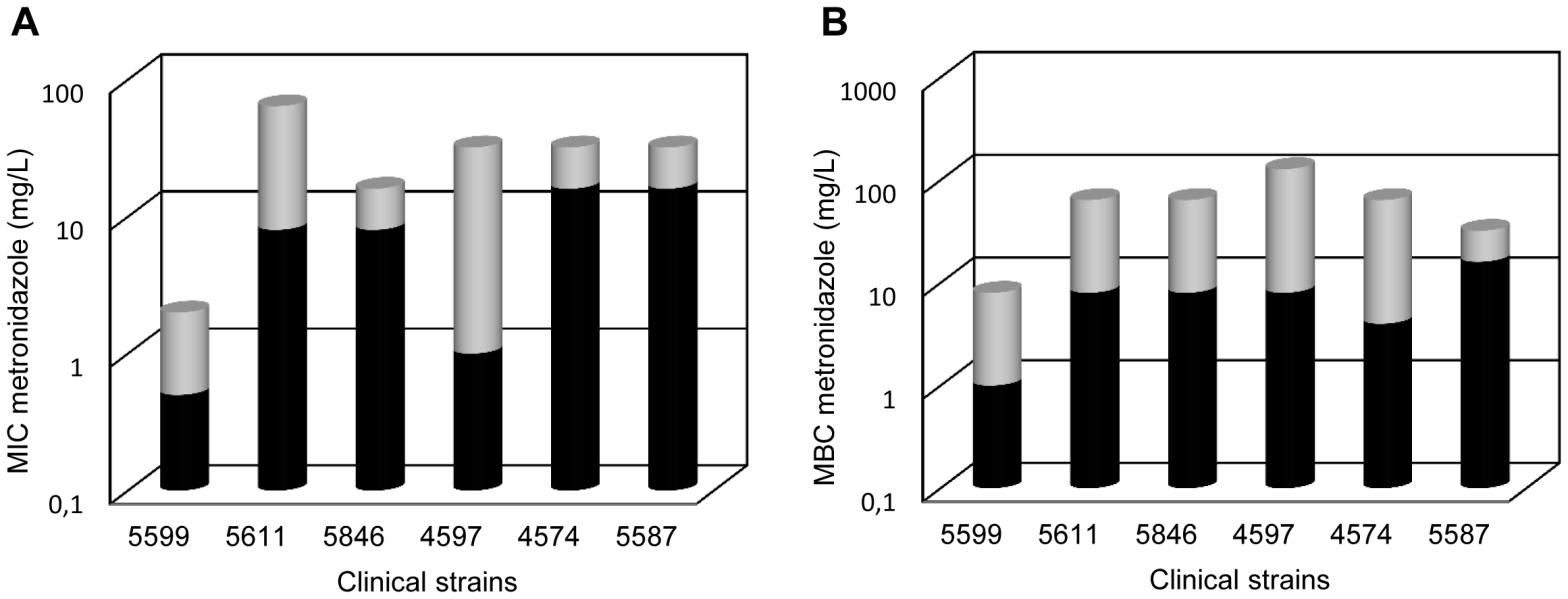
CORM-2 lowers MIC and MBC of metronidazole for *H. pylori* clinical isolates. (**A**) MIC of metronidazole determined for each *H pylori* clinical isolate in the absence (grey) and in the presence of CORM-2 (black). Strains 5846 and 5587 were treated 50 mg/CORM-2, strains 5611 and 4574 exposed to 100 mg/L CORM-2, and strains 5599 and 4597 submitted to 150 mg/L CORM-2. (**B**) MBC of metronidazole for each *H pylori* clinical isolates, in the absence (grey) and in the presence of CORM-2 (black). Strains 5846 and 5587 were treated 200 mg/CORM-2, and strains 5611, 4574, 5599 and 4597 exposed to 150 mg/L CORM-2. For each strain, metronidazole was combined with a CORM-2 concentration below the MIC/MBC of the CORM-2 alone. In all cases, values representing the median of five biological samples were significantly different (p<0.05 in Mann Whitney *t* test).

The effect of combining metronidazole with CORM-2 was also tested for *H. pylori* clinical isolates. At least, a two-fold decrease of the MIC for metronidazole was observed in all cases, with the highest metronidazole resistant strains exhibiting the more significant drop of the MIC and MBC values (Fig. 6). For example, the clinical isolate 5611 (MIC_metronidazole_ = 64 mg/L) become susceptible (MIC for metronidazole = 8 mg/L) when the metronidazole was administrated together with 100 mg/L CORM-2 (Tables S3 and S4).

### CORM Treated *H. pylori* Compromises Bacterial Survival in Macrophages

**Figure 7:**
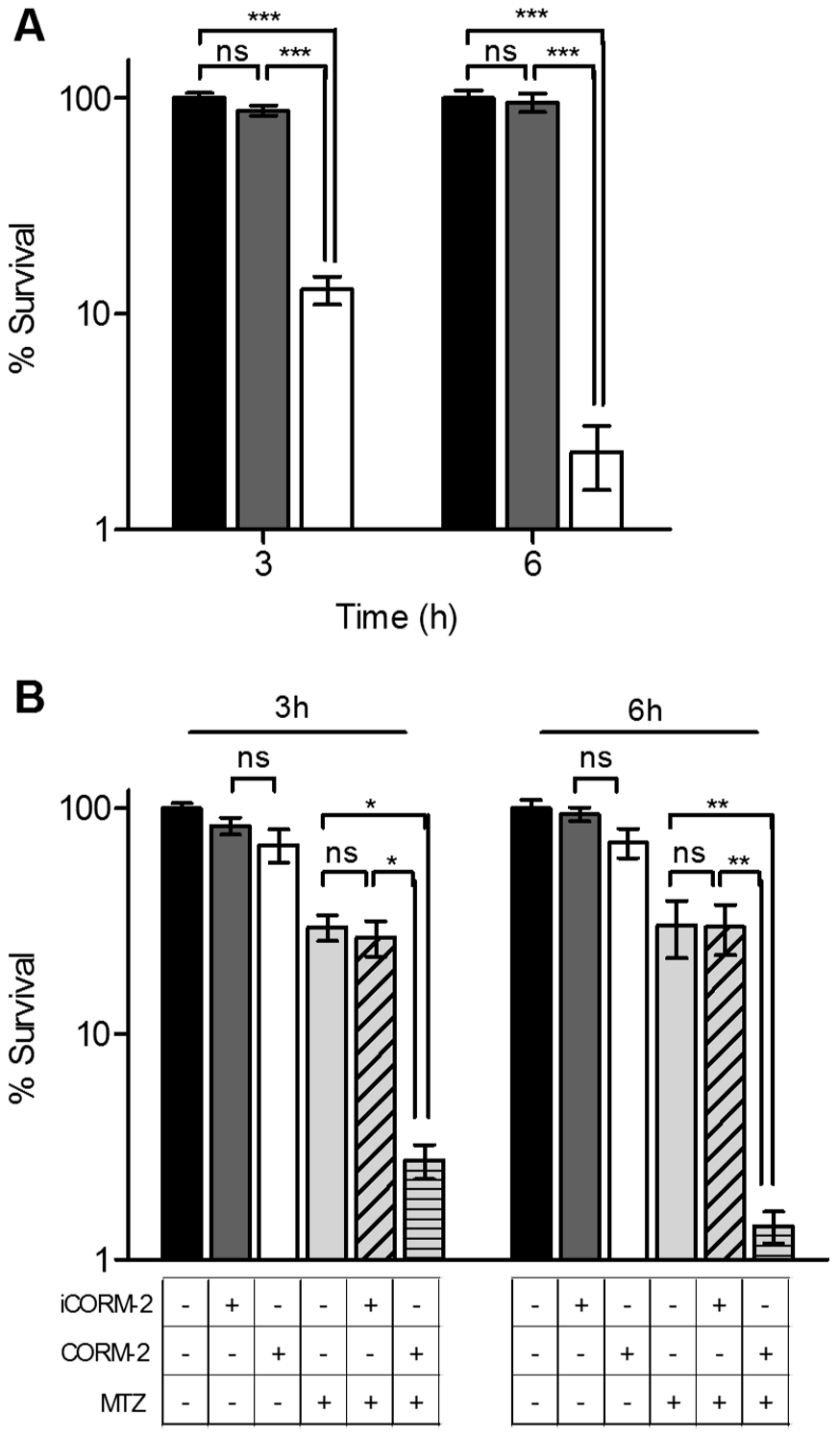
*H. pylori* is more susceptible to macrophage killing when treated with CORM-2. Macrophages RAW264.7, pre-activated with IFN-γ (0.3 µg/mL) for 12 h, were infected with *H. pylori* (MOI ∼100). In (**A**) *H. pylori* untreated (black), treated with 400 mg/L iCORM-2 (grey) or with 200 mg/L CORM-2 (white) was used. For (**B**) *H. pylori* not exposed (black), submitted to 200 mg/L iCORM-2 (dark grey), 100 mg/L CORM-2 (white), 1.5 mg/L MTZ (light grey), MTZ plus iCORM-2 (grey, diagonal strips), and MTZ combined with 100 mg/L CORM-2 (grey, horizontal strips) was assayed. In all cases, bacterial survival was determined after 3 and 6 h of infection, and results are the average of five biological samples analysed in triplicate. Error bars represent SE. *p<0.05, **p<0.01, ns - non significant (Two-way ANOVA and Bonferroni test).

Since *H. pylori* is known to activate the innate immune system we have evaluated the effect of CORM-2 upon *H. pylori* infection of murine macrophages. *H. pylori* cells unexposed or exposed to iCORM-2 and CORM-2 were incubated with activated RAW264.7 macrophages and their viability evaluated. For similar viable bacterial loads, the iCORM-2-treated *H. pylori* was as resistant to macrophages as untreated bacterial cells. On the contrary, the survival of the CORM-2-treated *H. pylori* was approximately 98% lower in comparison to cells exposed to iCORM-2 (Fig. 7A).

### Combined CORM-2-Metronidazole Further Reduces Survival of *H. pylori* in Macrophages

Given that the combination of CORM-2 with metronidazole reduced the *in* *vitro* viability of *H. pylori*, we next determined the survival of the double-treated *H. pylori* when in contact with activated macrophages. While no significant alteration was seen when infecting macrophages with *H. pylori* treated with CORM-2 (100 mg/L) or iCORM-2 (200 mg/L) alone, a small decrease in the survival (∼30%) was observed for cells exposed to metronidazole or metronidazole plus the iCORM-2 (Fig. 7B). However, treatment of *H. pylori* with CORM-2 (100 mg/L) combined with metronidazole (1.5 mg/L) exhibited a ∼98% decrease in bacterial count upon macrophage infection. This allows concluding that the co-administration of metronidazole-CORM-2 renders *H. pylori* more susceptible to macrophage killing.

## Discussion

The current work reveals that CORMs are effective against *H. pylori* with CORM-2 being more effective than CORM-3. This difference is likely to originate from the different reactivity and physical-chemical properties (e.g. hydrophobicity, polarity, H-bonding ability, etc.) imparted to both complexes by their outer coordination spheres [12]. In particular, CORM-2 is more hydrophobic than CORM-3, which may favour the interaction of CORM-2 with the medium and the bacterial cells. We also observed that Ru^2+^ complexes are not toxic as iCORM-2 does not decrease *H. pylori* viability, indicating that the bactericidal action of CORM-2 relies on inhibition of the bacterial components by CO. In agreement, inhibition of *H. pylori* growth by CORM-2 is accompanied by a decrease in the rate of cellular oxygen consumption due to the binding of CORM-2-derived CO to the *H. pylori* terminal oxidase. Similar findings were reported for other bacteria [16], [25],[26]. Moreover, CORM-2 impairs the urease activity, which is a nonheme target, most probably due to the ligation of CO to nickel for which CO has high affinity [27]. Due to the crucial role of urease in the persistence of *H. pylori* in the gastric niche [3], a urease inhibitor such as CORM-2 gains relevance as a novel way to control this infection. Moreover, and contrary to other potent urease inhibitors such as acetohydroxamic acid and fluorofamide [28], [29], these CORMs preserve their stability at low pH values [14], representing a clear advantage for *H. pylori* treatment as they must be active in the acidic environment of the stomach.

Although our data show that CORM-derived CO affected two metal containing enzymes, namely the heme containing terminal oxidases and Ni-containing urease, it is likely that these will not be the sole targets, since CO has a high affinity for metals and may bind other metal containing enzymes and proteins, including regulators like Fur or NikR.

Another interesting feature of CORM-2 is the apparent lack of alterations of the morphologic traits in *H. pylori* cells treated with this compound, revealing that CORM-2 does not induce the formation of coccoid forms, which in several cases are associated with development of *H. pylori* antibiotic resistance [30].

For *H. pylori*, approximately 200 mg/L (∼400 µM) CORM-2 was required to decrease viability by more than 99.99%, while for *E. coli*, *S. aureus* and *P. aeruginosa* the inhibitory concentration ranged from 100–500 µM [15], [16]. Although the concentrations required to kill a pathogen are certainly related with the type of microorganism, the growth medium and the experimental conditions also contribute to the amounts required in each case. The hitherto available data indicates that bacteria cultivated in minimal media are killed by concentrations of CORMs that are lower than those required to eliminate bacteria cultured in rich media [15]. The lack of effect observed on *H. pylori* when using ∼100 µM CORM-2 or CORM-3 is consistent with the failure also reported for *C. jejuni* grown in rich medium and exposed to 100 µM CORM-3 [25]. Like *Campylobacter jejuni, H. pylori* is a slow growth pathogen that requires rich medium to proliferate *in* *vitro*. Therefore, only concentrations above 300 µM were effective for *H. pylori*.

In spite of the high concentrations of CORM-2 used here to kill *H. pylori*, it is worth noting that they were determined under *in* *vitro* conditions. These are likely very different from those expected at the *in* *vivo* environment, as several factors will influence the amounts required to eradicate *H. pylori*, which include the acidity and oxygen conditions of the milieu at the site of infection. Nevertheless, CORM-2 and CORM-3 used in concentrations up to 500 µM are not toxic to mammalian cells [13], [16], [31]. Moreover, CORMs seem to be biologically effective as treatment of mice with an intraperitoneal injection significantly decreased the *P. aeruginosa* counts in spleen and the mortality of mice without any sign of toxicity [16].

Treatment failures are common in patients infected with *H. pylori* strains resistant to metronidazole, which is one of the antibiotics currently used in therapy of *H. pylori* [5]. We proved that metronidazole-resistant clinical strains are eliminated by CORM-2. Moreover, combination of CORM-2 with metronidazole is more effective at killing *H. pylori* than either drug alone, and enhances the killing promoted by macrophages. Furthermore, the present results suggest that CORM-2 has the potential to eradicate amoxicillin and clarithromycin resistant *H. pylori* strains, as the combination of both drugs also decreased the MIC for amoxicillin and clarithromycin against *H. pylori* 26695 reference strain. Although the resistance to metronidazole can be overcome by increasing the dose and duration of the therapy, it may lead to intolerable side effects [5]. In this way, the use of CORM-2 as co-adjuvant may represent an alternative treatment.

Recently, it was reported that although the ruthenium-based carbonyl ALF492 does not have an antiparasitic effect, it enhances the action of the antimalarial drug artesunate [32]. Also, CORM-2 combined with tobramycin, a drug used to treat *P. aeruginosa* lung infections, seems to prevent biofilm formation [33]. However, some of the *P. aeruginosa* clinical isolates tested were not susceptible to CORM-2 suggesting that it may not work for all *P. aeruginosa* infections. On the contrary, all *H. pylori* clinical strains herein examined were susceptible to CORM-2, including those resistant to metronidazole and to clarithromycin.

Treatment of *H. pylori* infections with CORMs may have further impact that is not strictly related with the elimination of the bacterium. One of the hallmarks of *H. pylori* infection is the induction of a state of chronic inflammation in the gastric mucosa, which *H. pylori* exploits to promote epithelial erosion and to acquire essential nutrients. CO is produced by the human HO-1 enzyme as a natural mechanism of controlling and reducing the inflammatory response. Application of exogenous CO also protects against inflammation and CORMs have been used as therapies to decrease undesired inflammatory responses [10], [34]. Hence, the use of CORMs against *H. pylori* may constitute a source of CO release in the gastric lumen potentially contributing to the decrease of mucosal inflammation, which is a major cause for the development of malignant lesions. Indeed, it has been shown that CO delivered by ALF186 ([Mo(CO)3(histidinato)]Na) is biologically active in the stomach providing protection against the inflammation and gastric ulcer caused by indomethacin [35]. Most likely relevant to the same issue is the fact that ruthenium CORMs like CORM-3 and ALF492 induce HO-1, which results in a reinforcement of their anti-inflammatory action [32]. Besides contributing to cytoprotection [9], CORMs may also promote reinforcement of the innate immune system as we show that CORM-2-treated *H. pylori* has a very low survival in macrophages.

In conclusion, this work provides the first evidence that CORMs act as antimicrobials either alone or in combination with antibiotics currently used to *H. pylori*. This is important data since chronic colonization with *H. pylori* antibiotic-resistant strains is difficult to eradicate and combined therapies are among the more effective means to combat resistant strains.

## Supporting Information

Figure S1 **Effect of metronidazole on** ***H. pylori*** **viability.** *H. pylori* 26695, grown as described in Material and Methods, was treated with 1.5 and 2 mg/L metronidazole (black and white squares, respectively) or left untreated (black circles). Cell viability was analyzed at the indicated times by determining CFU/mL. Values are average of two biological samples with the respective SE.
(TIF)

Figure S2 **iCORM-2 has no effect on** ***H. pylori*** **viability.** *H. pylori* 26695 left untreated (black bar) and after exposure to 200, 300 and 400 mg/L iCORM-2 (dark grey, white and light grey bars, respectively). Cell viability was determined as described in Material and Methods. Values represent the average of three biological samples with SE.
(TIF)

Table S1 **MIC and MBC of CORM-2 (mg/L) and metronidazole (mg/L) for the reference strain 26695 and the indicated clinical isolates of** ***H. pylori.***
(DOCX)

Table S2 **MIC and MBC (mg/L) of metronidazole (MTZ), clarithromycin (CH) and amoxicillin (AMX) for** ***H. pylori*** **26695 determined in the presence of sub-lethal doses of CORM-2.**
(DOCX)

Table S3 **MIC of metronidazole (mg/L) combined with sub-lethal doses of CORM-2 (mg/L) for clinical isolates of** ***H. pylori.***
(DOCX)

Table S4 **MBC of metronidazole (mg/L) combined with sub-lethal doses of CORM-2 (mg/L) for clinical isolates of** ***H. pylori.***
(DOCX)

Protocol S1 **Growth conditions for viability assays.** To determine the susceptibility of *H. pylori* to CORMs, cells cultured on HBA plates for 24 h were used to inoculate 10 mL of BHI-FCS liquid media contained in 25 cm^3^ cell culture flasks (Nunc) at an optical density at 600 nm (OD_600_) ∼0.05. After 16 h, these cultures were used as starter cultures to inoculate *H. pylori* in 10 mL BHI-βCD at an OD_600_ ∼0.05. At this point, CORM-3, CORM-2, iCORM-2 and/or metronidazole were added and growth was monitored by recording the OD_600_ for the next 20 h. At selected times, the number of viable cells was evaluated by measuring the colony-forming unit per millilitre (CFU/mL) formed upon plating serial dilutions on HBA plates, which were incubated three days.
(DOC)

Protocol S2 **Determination of MIC and MBC.** Starting cultures of *H. pylori* 26695 and clinical isolates, prepared as described above, were used to inoculate fresh BHI-βCD medium to an OD_600_ of ∼0.05, and aliquots of 1.2 mL were distributed in 24-well plates (Sarstedt). For each antibiotic the following range of concentrations were used with increasing doubling concentrations: metronidazole 0.5–256 mg/L, amoxicillin 0.001–0.250 mg/L and clarithromycin 0.001–0.250 mg/L. The range of CORM-3 and CORM-2 concentrations varied from 50 to 600 mg/L, with 50 mg/L intervals. After microaerobic incubation for 72 h, at 37°C and 90 rpm, MICs were determined by reading the OD_600_. For the MBCs determination, 10 µL of each culture was then plated on HBA medium and incubated for another 72 h and the lowest concentration that prevented formation of colonies was considered the MBC.
(DOC)

Protocol S3 **Preparation of** ***H. pylori*** **cellular suspension for oxygen consumption assays and urease activity.** To determine the rate of oxygen consumption, starting cultures of *H. pylori* 26695, prepared as described above, were used to inoculate fresh BHI-βCD medium to an OD_600_ of ∼0.05. Then, after 15 h, cultures were harvested by centrifugation (5 min, 12000×g, 4°C), washed and resuspended in 10 mM potassium phosphate buffer (pH 7.0). This cellular suspension was further incubated for 5 min with CORM-2 (25 mg/L), iCORM-2 (50 mg/L) or left untreated, and used for oxygen consumption assays. To measure urease activity in *H. pylori*, starting cultures, prepared as described above, were used to inoculate fresh BHI-βCD medium to an OD_600_ of ∼0.05. *H. pylori* was grown for 15 h, in BHI-βCD in the absence and in the presence of 200 mg/L CORM-2 or 400 mg/L iCORM-2. Before analysis, cultures were diluted, to achieve a final concentration of 1×10^8^ CFU/mL (OD_600_ ∼0.1). The effect of CORM-2 was also evaluated in cellular suspensions *in* *vitro*, treated for 15 min with increasing concentrations of CORM-2. In this case, a *H. pylori* suspension was prepared, using cells grown for 24 h on HBA plates, in PBS at 2×10^8^ CFU/mL (OD_600_ ∼0.2), and treated with CORM-2 (0, 2.5, 5, 12.5, 25, 50, 100 and 200 mg/L) for 15 min.
(DOCX)

Protocol S4 **Macrophages Experiments.** The Dulbecco’s modified Eagle’s medium used to cultivate macrophages contains 4.5 g/L glucose and 110 mg/mL sodium pyruvate (DMEM glutamax™, Gibco-Invitrogen) and is supplemented with 10% FCS, 70 U/mL penicillin and 70 µg/mL streptomycin (Gibco-Invitrogen). Cultures of *H. pylori* 26695, grown as described above for the viability assays, in the presence of CORM-2, iCORM-2 and/or metronidazole for 15 h, were washed three times with PBS (pH 7.4) and resuspended in infection medium containing DMEM glutamax™ supplemented with 10% FCS, without addition of antibiotics, at an initial bacterial content of ∼5×10^8^ CFU/mL. Bacterial suspensions (100 µL) were used to infect macrophages cultured in infection medium, at a multiplicity of infection (MOI) of ∼100. After incubation for 3 and 6 h, at 37°C and 5% CO_2_, each well was scraping to release adherent cells and resuspended in BHI medium; viable bacterial cells were then evaluated by plating serial dilutions onto HBA plates, which were incubated for 3 days. The values were normalized to the initial value of CFU/mL, i.e the CFU of the culture immediately before been used to infect macrophages, and the survival percentage determined by dividing the number of colonies of treated cultures by those of untreated cultures.
(DOCX)
