# Supplementary figures and images for: The Bactericidal Activity of Carbon Monoxide–Releasing Molecules against Helicobacter pylori
Source: PLoS One. 2013 Dec 26;8(12):e83157. doi: 10.1371/journal.pone.0083157 (PMC3873287; doi:10.1371/journal.pone.0083157)

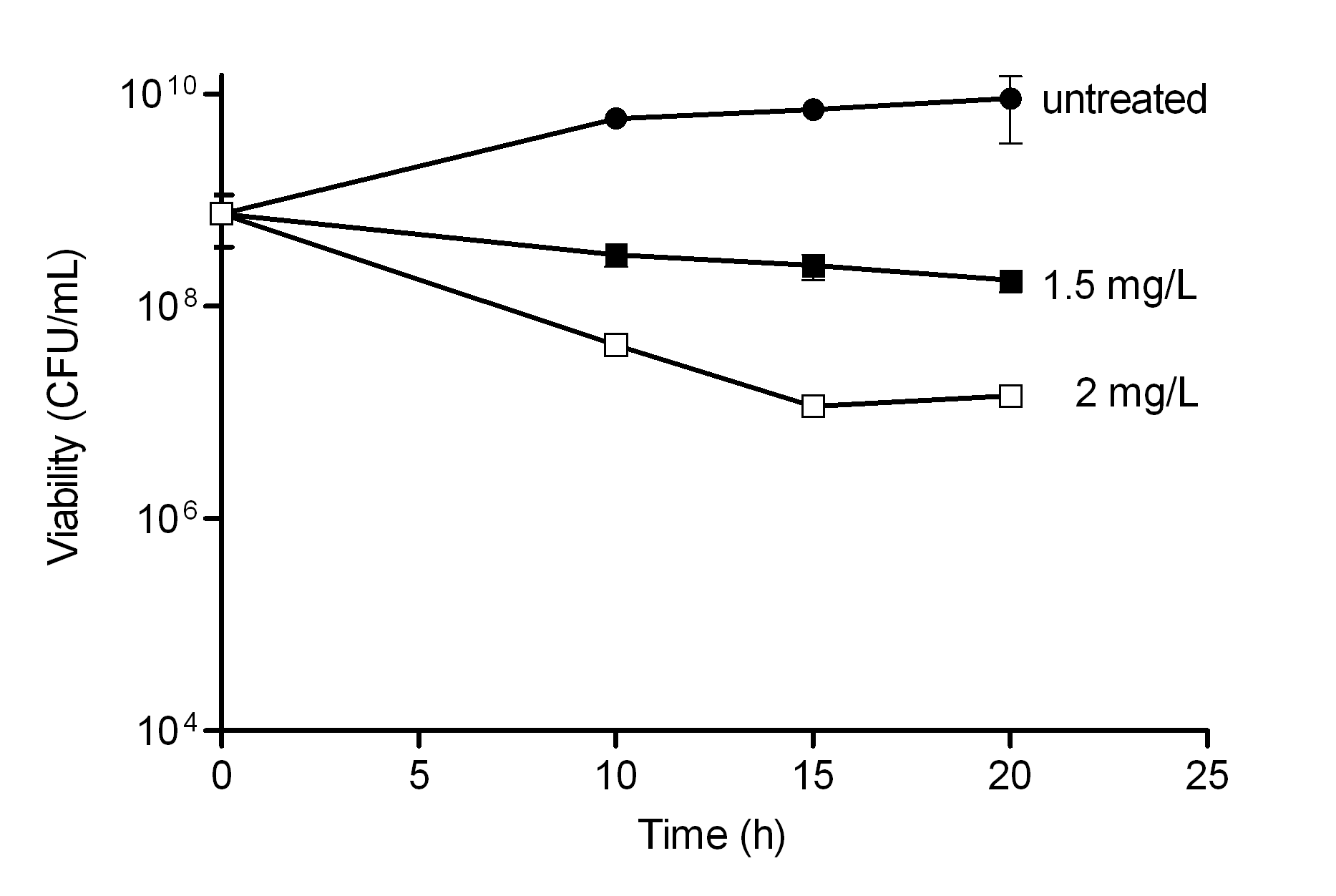

Supplement: Figure S1 — Effect of metronidazole on H. pylori viability. H. pylori 26695, grown as described in Material and Methods, was treated with 1.5 and 2 mg/L metronidazole (black and white squares, respectively) or left untreated (black circles). Cell viability was analyzed at the indicated times by determining CFU/mL. Values are average of two biological samples with the respective SE. (TIF) [file pone.0083157.s001.tif]

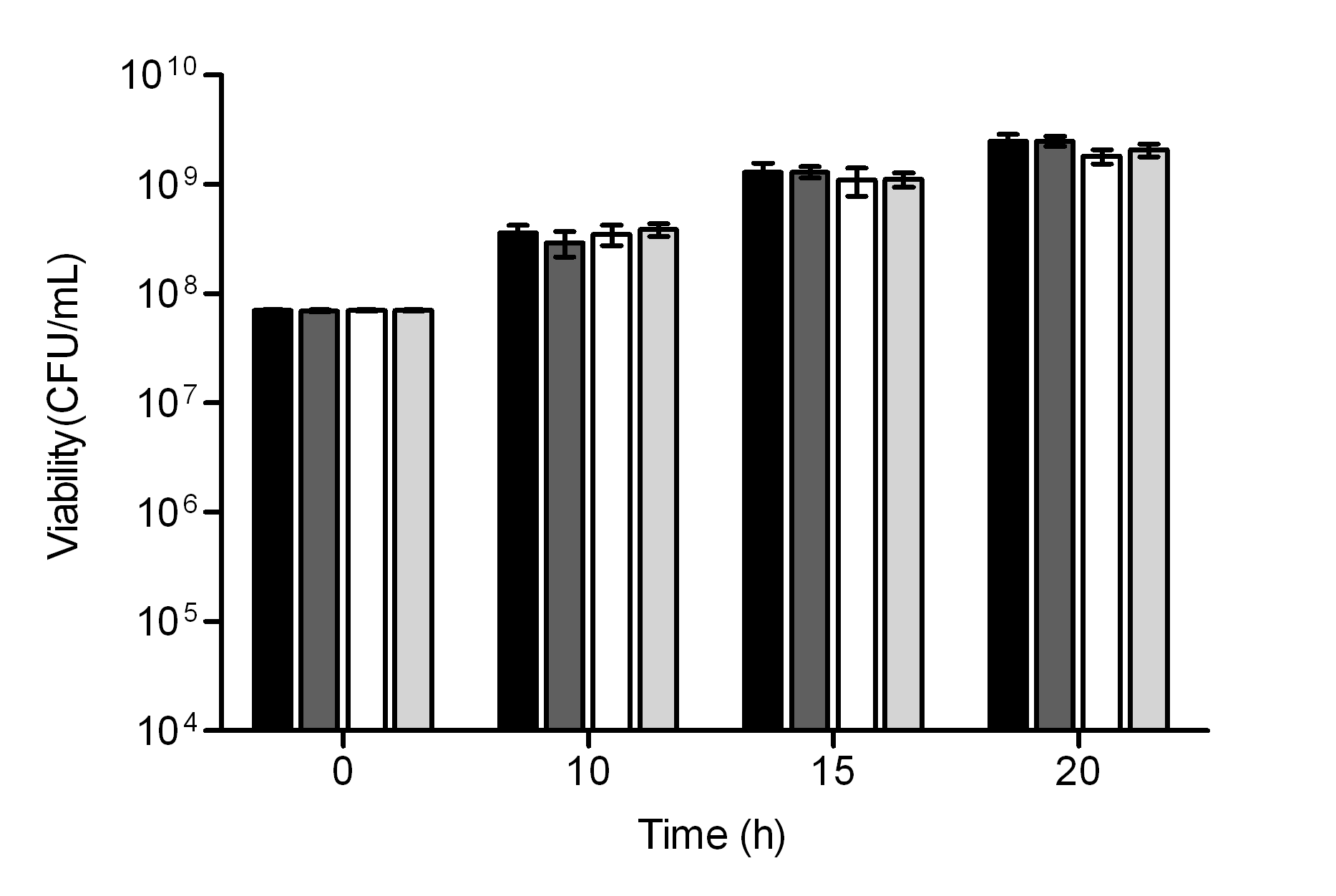

Supplement: Figure S2 — iCORM-2 has no effect on H. pylori viability. H. pylori 26695 left untreated (black bar) and after exposure to 200, 300 and 400 mg/L iCORM-2 (dark grey, white and light grey bars, respectively). Cell viability was determined as described in Material and Methods. Values represent the average of three biological samples with SE. (TIF) [file pone.0083157.s002.tif]
