# Supplementary material for: The Bactericidal Activity of Carbon Monoxide–Releasing Molecules against Helicobacter pylori
Source: PLoS One. 2013 Dec 26;8(12):e83157. doi: 10.1371/journal.pone.0083157 (PMC3873287; doi:10.1371/journal.pone.0083157)
Supplement: Table S1 — MIC and MBC of CORM-2 (mg/L) and metronidazole (mg/L) for the reference strain 26695 and the indicated clinical isolates of H. pylori. (DOCX) [file pone.0083157.s003.docx]

**Table S1.** MIC and MBC of CORM-2 (mg/L) and metronidazole (mg/L) for the reference strain 26695 and the indicated clinical isolates of *H. pylori.*

|  | **CORM-2** | | | **Metronidazole*** | | |
| --- | --- | --- | --- | --- | --- | --- |
| **Strain** | **MIC** | **MBC** | **MBC/MIC** | **MIC** | **MBC** | **MBC/MIC** |
| **26695** | 200 | 250 | 1.3 | 8 | 16 | 2 |
| **5599** | 200 | 200 | 1.0 | 2 | 8 | 4 |
| **5611** | 150 | 200 | 1.3 | 64 | 64 | 1 |
| **5846** | 100 | 150 | 1.5 | 16 | 64 | 4 |
| **4597** | 200 | 200 | 1.0 | 32 | 128 | 4 |
| **4574** | 150 | 200 | 1.3 | 32 | 64 | 2 |
| **5587** | 100 | 150 | 1.5 | 32 | 32 | 1 |

*S: Sensitive (MIC<8 mg/L)

R: Resistant (MIC>8 mg/L)
