# Supplementary material for: The Bactericidal Activity of Carbon Monoxide–Releasing Molecules against Helicobacter pylori
Source: PLoS One. 2013 Dec 26;8(12):e83157. doi: 10.1371/journal.pone.0083157 (PMC3873287; doi:10.1371/journal.pone.0083157)
Supplement: Table S2 — MIC and MBC (mg/L) of metronidazole (MTZ), clarithromycin (CH) and amoxicillin (AMX) for H. pylori 26695 determined in the presence of sub-lethal doses of CORM-2. (DOCX) [file pone.0083157.s004.docx]

**Table S2.** MIC and MBC (mg/L) of metronidazole (MTZ), clarithromycin (CH) and amoxicillin (AMX) for *H. pylori* 26695 determined in the presence of sub-lethal doses of CORM-2.

| **CORM-2** | | **MIC** | | | | **MBC** | | | | **MBC/MIC** | | | |
| --- | --- | --- | --- | --- | --- | --- | --- | --- | --- | --- | --- | --- | --- |
|  | **MTZ** | | **CH** | **AMX** | **MTZ** | | **CH** | **AMX** | **MTZ** | | **CH** | **AMX** |  |
| 0 | 8 | | 0.016 | 0.125 | 16 | | 0.032 | 0.25 | 2 | | 2 | 2 |  |
| 100 | 4 | | 0.008 | 0.064 | 16 | | 0.008 | 0.125 | 4 | | 1 | 2 |  |
| 150 | 2 | | 0.004 | 0.064 | 4 | | 0.008 | 0.064 | 2 | | 2 | 1 |  |
