# Supplementary material for: The Bactericidal Activity of Carbon Monoxide–Releasing Molecules against Helicobacter pylori
Source: PLoS One. 2013 Dec 26;8(12):e83157. doi: 10.1371/journal.pone.0083157 (PMC3873287; doi:10.1371/journal.pone.0083157)
Supplement: Table S3 — MIC of metronidazole (mg/L) combined with sub-lethal doses of CORM-2 (mg/L) for clinical isolates of H. pylori. (DOCX) [file pone.0083157.s005.docx]

**Table S3.** MIC of metronidazole (mg/L) combined with sub-lethal doses of CORM-2 (mg/L) for clinical isolates of *H. pylori.*

|  | **MIC_metronidazole_** | | | | | |  |
| --- | --- | --- | --- | --- | --- | --- | --- |
| **Strain**  **CORM-2** | **5599** | **5611** | **5846** | **4597** | **4574** | **5587** | |
| 0 | 2 | 64 | 16 | 32 | 32 | 32 | |
| 50 | 2 | 16 | 8 | 32 | 32 | 16 | |
| 100 | 1 | 8 | - | 4 | 16 | - | |
| 150 | 0.5 | - | - | 1 | - | - | |
