# Supplementary material for: The Bactericidal Activity of Carbon Monoxide–Releasing Molecules against Helicobacter pylori
Source: PLoS One. 2013 Dec 26;8(12):e83157. doi: 10.1371/journal.pone.0083157 (PMC3873287; doi:10.1371/journal.pone.0083157)
Supplement: Protocol S1 — Growth conditions for viability assays. To determine the susceptibility of H. pylori to CORMs, cells cultured on HBA plates for 24 h were used to inoculate 10 mL of BHI-FCS liquid media contained in 25 cm3 cell culture flasks (Nunc) at an optical density at 600 nm (OD600) ∼0.05. After 16 h, these cultures were used as starter cultures to inoculate H. pylori in 10 mL BHI-βCD at an OD600 ∼0.05. At this point, CORM-3, CORM-2, iCORM-2 and/or metronidazole were added and growth was monitored by recording the OD600 for the next 20 h. At selected times, the number of viable cells was evaluated by measuring the colony-forming unit per millilitre (CFU/mL) formed upon plating serial dilutions on HBA plates, which were incubated three days. (DOC) [file pone.0083157.s007.doc]

**Protocol S1. Growth conditions for viability assays.**

To determine the susceptibility of *H. pylori* to CORMs, cells cultured on HBA plates for 24 h were used to inoculate 10 mL of BHI-FCS liquid media contained in 25 cm^3^ cell culture flasks (Nunc) at an optical density at 600 nm (OD_600_) ~0.05. After 16 h, these cultures were used as starter cultures to inoculate *H. pylori* in 10 mL BHI-βCD at an OD_600_ ~0.05. At this point, CORM-3, CORM-2, iCORM-2 and/or metronidazole were added and growth was monitored by recording the OD_600_ for the next 20 h. At selected times, the number of viable cells was evaluated by measuring the colony-forming unit per millilitre (CFU/mL) formed upon plating serial dilutions on HBA plates, which were incubated three days.
