# Supplementary material for: The Bactericidal Activity of Carbon Monoxide–Releasing Molecules against Helicobacter pylori
Source: PLoS One. 2013 Dec 26;8(12):e83157. doi: 10.1371/journal.pone.0083157 (PMC3873287; doi:10.1371/journal.pone.0083157)
Supplement: Protocol S2 — Determination of MIC and MBC. Starting cultures of H. pylori 26695 and clinical isolates, prepared as described above, were used to inoculate fresh BHI-βCD medium to an OD600 of ∼0.05, and aliquots of 1.2 mL were distributed in 24-well plates (Sarstedt). For each antibiotic the following range of concentrations were used with increasing doubling concentrations: metronidazole 0.5–256 mg/L, amoxicillin 0.001–0.250 mg/L and clarithromycin 0.001–0.250 mg/L. The range of CORM-3 and CORM-2 concentrations varied from 50 to 600 mg/L, with 50 mg/L intervals. After microaerobic incubation for 72 h, at 37°C and 90 rpm, MICs were determined by reading the OD600. For the MBCs determination, 10 µL of each culture was then plated on HBA medium and incubated for another 72 h and the lowest concentration that prevented formation of colonies was considered the MBC. (DOC) [file pone.0083157.s008.doc]

**Protocol S2. Determination of MIC and MBC.**

Starting cultures of *H. pylori* 26695 and clinical isolates, prepared as described above, were used to inoculate fresh BHI-βCD medium to an OD_600_ of ~0.05, and aliquots of 1.2 mL were distributed in 24-well plates (Sarstedt). For each antibiotic the following range of concentrations were used with increasing doubling concentrations: metronidazole 0.5-256 mg/L, amoxicillin 0.001-0.250 mg/L and clarithromycin 0.001-0.250 mg/L. The range of CORM-3 and CORM-2 concentrations varied from 50 to 600 mg/L, with 50 mg/L intervals. After microaerobic incubation for 72 h, at 37ºC and 90 rpm, MICs were determined by reading the OD_600_. For the MBCs determination, 10 µL of each culture was then plated on HBA medium and incubated for another 72 h and the lowest concentration that evented formation of colonies was considered the MBC.
