# Supplementary material for: The Bactericidal Activity of Carbon Monoxide–Releasing Molecules against Helicobacter pylori
Source: PLoS One. 2013 Dec 26;8(12):e83157. doi: 10.1371/journal.pone.0083157 (PMC3873287; doi:10.1371/journal.pone.0083157)
Supplement: Protocol S3 — Preparation of H. pylori cellular suspension for oxygen consumption assays and urease activity. To determine the rate of oxygen consumption, starting cultures of H. pylori 26695, prepared as described above, were used to inoculate fresh BHI-βCD medium to an OD600 of ∼0.05. Then, after 15 h, cultures were harvested by centrifugation (5 min, 12000×g, 4°C), washed and resuspended in 10 mM potassium phosphate buffer (pH 7.0). This cellular suspension was further incubated for 5 min with CORM-2 (25 mg/L), iCORM-2 (50 mg/L) or left untreated, and used for oxygen consumption assays. To measure urease activity in H. pylori, starting cultures, prepared as described above, were used to inoculate fresh BHI-βCD medium to an OD600 of ∼0.05. H. pylori was grown for 15 h, in BHI-βCD in the absence and in the presence of 200 mg/L CORM-2 or 400 mg/L iCORM-2. Before analysis, cultures were diluted, to achieve a final concentration of 1×10^8^ CFU/mL (OD600 ∼0.1). The effect of CORM-2 was also evaluated in cellular suspensions in vitro, treated for 15 min with increasing concentrations of CORM-2. In this case, a H. pylori suspension was prepared, using cells grown for 24 h on HBA plates, in PBS at 2×10^8^ CFU/mL (OD600 ∼0.2), and treated with CORM-2 (0, 2.5, 5, 12.5, 25, 50, 100 and 200 mg/L) for 15 min. (DOCX) [file pone.0083157.s009.docx]

**Protocol S3. Preparation of *H. pylori* cellular suspension for oxygen consumption assays and urease activity.**

To determine the rate of oxygen consumption, starting cultures of *H. pylori* 26695, prepared as described above, were used to inoculate fresh BHI-βCD medium to an OD_600_ of ~0.05. Then, after 15 h, cultures were harvested by centrifugation (5 min, 12000 × g, 4 °C), washed and resuspended in 10 mM potassium phosphate buffer (pH 7.0). This cellular suspension was further incubated for 5 min with CORM-2 (25 mg/L), iCORM-2 (50 mg/L) or left untreated, and used for oxygen consumption assays.

To measure urease activity in *H. pylori*, starting cultures, prepared as described above, were used to inoculate fresh BHI-βCD medium to an OD_600_ of ~0.05. *H. pylori* was grown for 15 h, in BHI-βCD in the absence and in the presence of 200 mg/L CORM-2 or 400 mg/L iCORM-2. Before analysis, cultures were diluted, to achieve a final concentration of 1 x 10^8^ CFU/mL (OD_600_ ~0.1). The effect of CORM-2 was also evaluated in cellular suspensions *in vitro*, treated for 15 min with increasing concentrations of CORM-2. In this case, a *H. pylori* suspension was prepared, using cells grown for 24 h on HBA plates, in PBS at 2 x 10^8^ CFU/mL (OD_600_ ~0.2), and treated with CORM-2 (0, 2.5, 5, 12.5, 25, 50, 100 and 200 mg/L) for 15 min.
