# Supplementary material for: The Bactericidal Activity of Carbon Monoxide–Releasing Molecules against Helicobacter pylori
Source: PLoS One. 2013 Dec 26;8(12):e83157. doi: 10.1371/journal.pone.0083157 (PMC3873287; doi:10.1371/journal.pone.0083157)
Supplement: Protocol S4 — Macrophages Experiments. The Dulbecco’s modified Eagle’s medium used to cultivate macrophages contains 4.5 g/L glucose and 110 mg/mL sodium pyruvate (DMEM glutamax™, Gibco-Invitrogen) and is supplemented with 10% FCS, 70 U/mL penicillin and 70 µg/mL streptomycin (Gibco-Invitrogen). Cultures of H. pylori 26695, grown as described above for the viability assays, in the presence of CORM-2, iCORM-2 and/or metronidazole for 15 h, were washed three times with PBS (pH 7.4) and resuspended in infection medium containing DMEM glutamax™ supplemented with 10% FCS, without addition of antibiotics, at an initial bacterial content of ∼5×10^8^ CFU/mL. Bacterial suspensions (100 µL) were used to infect macrophages cultured in infection medium, at a multiplicity of infection (MOI) of ∼100. After incubation for 3 and 6 h, at 37°C and 5% CO2, each well was scraping to release adherent cells and resuspended in BHI medium; viable bacterial cells were then evaluated by plating serial dilutions onto HBA plates, which were incubated for 3 days. The values were normalized to the initial value of CFU/mL, i.e the CFU of the culture immediately before been used to infect macrophages, and the survival percentage determined by dividing the number of colonies of treated cultures by those of untreated cultures. (DOCX) [file pone.0083157.s010.docx]

**Protocol S4. Macrophages Experiments.**

The Dulbecco’s modified Eagle’s medium used to cultivate macrophages contains 4.5 g/L glucose and 110 mg/mL sodium pyruvate (DMEM glutamax^TM^, Gibco-Invitrogen) and is supplemented with 10% FCS, 70 U/mL penicillin and 70 µg/mL streptomycin (Gibco-Invitrogen).

Cultures of *H. pylori* 26695, grown as described above for the viability assays, in the presence of CORM-2, iCORM-2 and/or metronidazole for 15 h, were washed three times with PBS (pH 7.4) and resuspended in infection medium containing DMEM glutamax^TM^ supplemented with 10% FCS, without addition of antibiotics, at an initial bacterial content of ~5 x 10^8^ CFU/mL. Bacterial suspensions (100 µL) were used to infect macrophages cultured in infection medium, at a multiplicity of infection (MOI) of ~100. After incubation for 3 and 6 h, at 37°C and 5% CO_2_, each well was scraping to release adherent cells and resuspended in BHI medium; viable bacterial cells were then evaluated by plating serial dilutions onto HBA plates, which were incubated for 3 days. The values were normalized to the initial value of CFU/mL, i.e the CFU of the culture immediately before been used to infect macrophages, and the survival percentage determined by dividing the number of colonies of treated cultures by those of untreated cultures.
